# JACMP 2025–2029 and beyond

**DOI:** 10.1002/acm2.14448

**Published:** 2024-06-23

**Authors:** Michael Mills

**Affiliations:** ^1^ Radiation Oncology University of Louisville Louisville Kentucky USA

Any academic journal is a communication organ; it permits an author to transfer important, even critical information to all members of a profession. Such information is vetted by reviewers, associate editors and editors before dissemination. A journal therefore is a euphemism for structured relationships to accomplish truthful and meaningful content exchange. The JACMP ultimately serves patients by facilitating rapid communications that promote better quality healthcare within the context of professional publishing.

The common‐sense future of the JACMP is a convolution of the future trends of the medical physics profession with the future trends of academic publishing. As such, we should look at established and emerging trends of both of these spheres and spaces. Here are some of my thoughts and observations.

Present and future trends respecting the Medical Physics Profession:

1. The number of imaging procedures continue to grow at 2%−3% per year for the foreseeable future. This creates a baseline for the growth of need for imaging medial physicists.^1^
2. The worldwide business of medical imaging continues to grow at approximately 4%−5% per year. The economics of imaging may be growing faster than the growth in the number of procedures.^2^
3. The number of cancer patients continues to grow at around 2% per year.^3^
4. Due to the increasing complexity of practice and the increasing number of special procedures, I estimate the economics and professional work per patient is growing at around 2% per year in addition to the growth rate in the number of patients. However, this currently is an observation without supporting data.
5. There will likely be an undersupply of Qualified Medical Physicists as the growth in the number of CAMPEP accredited residency slots is not keeping pace with the growth of need for medical physics professionals.^4^
6. Clinical practicing medical physicists are working longer hours to keep pace with the workload, and many are working long hours and well into their retirement years to meet the challenge.^5^
7. Adding to the undersupply, ABR Board passing rates for medical physicists continue to lag well behind the passing rates for physicians trained in the radiology specialties. This creates an additional delay in the production of Qualified (Board Certified) Medical Physicists.^6^
8. There are significant communities within the United States and worldwide where the numbers of medical physicists are insufficient to serve the populations.^7^
9. Consequent to the above, salaries for medical physicists continue to grow at an average of 5%−6% per year. Job changers report median salary increases of about 12%.^8^

Summary of the above: Clinical medical physicists will be faced with snowballing work pressures. It will be increasingly more difficult for practicing medical physicists to find time to write clinical articles. While funded research article publications are usually supported by the granting agency, clinical physicists are usually responsible for paying the Article Publication Charge (APC) from personal funds. These deterrents may act to put limitations on the future numbers of JACMP submitted articles.

Present and future trends respecting Academic Publishing:

1. The trend among most journals is to eliminate print publication, convert to an open access publication model and change the business model to maintain profitability (if possible) within these new publishing realities. This movement has come more slowly than expected but is still considered inevitable.^9^, ^10^
2. The open access model results in greatly increased numbers of article downloads and citations. Articles published open access are 3.4 times more likely to be accessed, 1.4 times more likely to be cited and generate 4 times as much Altmetric attention as compared with subscription articles.^11^
3. While the elimination of print advertising has been challenging, publishers and societies are continuing to survive without the print advertising revenue.^12^
4. The Journal Impact Factor (JIF) has come under criticism as a means to assess the value of academic journal publications for the purpose of promotion and tenure. The San Francisco Declaration on Research Assessment is one example of this trend.^13^
5. A future trend among many universities is to ignore the JIF in favor of reporting the numbers of downloads and citations, considering the Altmetric scores and noting the social media presence of a journal article. The JIF would be used as a factor to decide what journals would be purchased by university libraries for employee, student and faculty access.^14^, ^15^
6. There is increased sensitivity to potential bias in all research literature respecting gender, ethnic and origins diversity.^16^

Summary of the above: The author community will probably trend toward selecting prominent and larger journals with a wide publication space. Journals with a wide publication space have high numbers of accesses, downloads and ultimately citations and social media presence. Authors may be less inclined to submit to small or new journals with limited presence in the community as the articles may have significantly fewer downloads and citations when evaluated. The JIF likely will not matter as much as it has in the past. Although this may be counterintuitive to many, the Journal Impact Factor is likely going away as an essential metric of article assessment. The value of an article often has little to do with the journal in which it is published. It is widely recognized that there are better metrics to support the assessment of articles for their impact in the community. Open access electronic publication greatly increases the potential for an article to be seen and cited. Authors will choose and demand robust and well‐established journals with established metrics of article downloads and citations. There will be resistance to publishing in novel or niche journals without dynamic article metrics. Diverse authors will expect and will receive a seat at the big table regardless of whatever barriers they previously experienced. In exchange, the journals will insist on publishing data and datasets free from bias (Table 1).

**TABLE 1:** Some statistical information regarding the JACMP.

Year	Editor‐in‐Chief	Deputy EIC	Deputy EIC	# Issues	# Articles	% Accepted	Journal impact
2000	Almond			4	19		Factor (update Year for Citations in Previous Two Years)
2001	Almond			4	27		
2002	Almond			4	36		
2003	McCullough			4	44		
2004	Mills	Solberg		4	31		
2005	Mills	Solberg		4	42		
2006	Mills	Solberg		4	40		
2007	Mills	Solberg		4	54		
2008	Starkschall	Solberg		4	67	52	1.216
2009	Starkschall	Solberg		4	88	49	1.225
2010	Starkschall	Solberg		4	106	51	1.008
2011	Starkschall	Solberg		4	108	48	1.291
2012	Starkschall	Solberg		6	135	48	0.959
2013	Mills	Solberg	Halvorsen	6	144	51	1.118
2014	Mills	Solberg	Halvorsen	6	193	51	1.111
2015	Mills	Solberg	Halvorsen	6	218	50	1.172
2016	Mills	Solberg	Halvorsen	6	248	48	1.444
2017	Mills	Solberg	Halvorsen	6	187	47	1.338
2018	Mills	Solberg	Halvorsen	6	262	45	1.301
2019	Mills	Solberg	Halvorsen	12	258	48	1.544
2020	Mills	Solberg	Halvorsen	12	316	61	1.679
2021	Mills	Solberg	Halvorsen	12	349	50	2.102
2022	Mills	Solberg	Richardson	12	347	56	2.243
2023	Mills	Solberg	Richardson	12	349	64	2.102
2024	Mills	Solberg	Richardson	12	–	–	–
https://www.bioxbio.com/journal/J‐APPL‐CLIN‐MED‐PHYS | Journal Impact Factor Reference							

Has the JACMP plateaued in growth?

Conflicting and Opposing Concepts to Ponder:

1. How can a profession grow in importance and stature when there are fewer professionals than are needed, trained, and available to do the work?
2. The logical progression of a professional is to master the content of the profession broadly, then acquire narrower and more detailed qualifications for specialty practice. However, medical physicists often train and acquire credentials for specialty practice at the beginning of their career, then seek to broaden their practice and knowledge into many areas outside the confines of their initial specialty.
3. The demands of scholarly inquiry likely will require the creation of new journals specific to new fields of endeavor. However, authors will need and demand larger journals with increasing publication space in order to maintain high metrics of downloads and citations for their articles.
4. While open access democratizes and levels access to literature and the ability to search ideas, the associated Article Publication Charge (APC) acts to restrict author access to these same journals.
5. Historically, print‐publication journals without double‐blind review (the reviewers know the identity of the authors) have been criticized for favorable bias respecting well‐established authors and programs. However, to maintain their status, such authors and programs may choose to publish in established open‐access journals with double‐blind peer review. Editors are noting increased interest among authors in double‐blind peer review journals.

The trend is for print‐publication journals to first transform to electronic‐only subscription publication, and then to open access publication. In response, mechanisms are emerging to cover the costs of open access publication so the burden of paying the APC does not fall on the authors. We are moving from a model where the author's work indirectly and financially benefits the owners and sponsors of journals to a model where the journals will be expected monetarily to break even; the advertisers will bypass the societies and reach the authors and readers directly, and the authors will no longer be responsible for paying the APC. This structure is well along and seems to be lagging open access by about 15 years. A similar lagging length of time exists for the movement to go from establishing open access to the realization of change in research assessment. However, as is now being seen, open access will ultimately require and make inevitable the abandoning of the JIF and the embracing of alternative metrics for article assessment respecting promotion and tenure.

What does all this mean for the AAPM and the JACMP? The JACMP is well‐positioned for the future. Depending on the criteria, the JACMP was either the first or second open access medical journal. It is robust, profitable, has experienced growth and may not have finished growing. The recent COVID lockdowns resulted in a sharp increase in the number of published articles in 2020 and 2021. When the lockdowns ended, the continuing underlying growth maintained the number of published articles at around 350 per year in 2022 and 2023. The JACMP appears to have plateaued, but my experience with the number of submissions suggests the growth has not ended and we are seeing submissions from programs and countries never seen before. I expect by the end of 2025, we will be publishing at about 400 papers per year.

If the AAPM creates new journals, this could impact the number of articles published in the JACMP. However, the current monthly number of submissions supports the opinion that JACMP may grow to recover its current numbers within the next 5 years, or by 2029, and then continue to grow. As long as clinical physicists require a communication organ to serve the worldwide cancer patient population, the JACMP should be there to meet that need.

## CONFLICT OF INTEREST STATEMENT

The author declares no conflicts of interest.
